# Advanced Time-Stepping Interpretation of Fly-Scan Continuous Rotation Synchrotron Tomography of Dental Enamel Demineralization

**DOI:** 10.1021/cbmi.3c00121

**Published:** 2024-02-08

**Authors:** Cyril Besnard, Ali Marie, Sisini Sasidharan, Shashidhara Marathe, Kaz Wanelik, Robert A. Harper, Christoph Rau, Richard M. Shelton, Gabriel Landini, Alexander M. Korsunsky

**Affiliations:** † Department of Engineering Science, University of Oxford, Parks Road, Oxford, Oxfordshire OX1 3PJ, United Kingdom; ‡ Diamond Light Source Ltd., Didcot, Oxfordshire OX11 0DE, United Kingdom; § School of Dentistry, University of Birmingham, 5 Mill Pool Way, Edgbaston, Birmingham, West Midlands B5 7EG, United Kingdom; ∥ Trinity College, University of Oxford, Broad St, Oxford, Oxfordshire OX1 3BH, United Kingdom

**Keywords:** Human carious enamel, Synchrotron, X-ray tomography, *In situ* demineralization, Microscopy

## Abstract

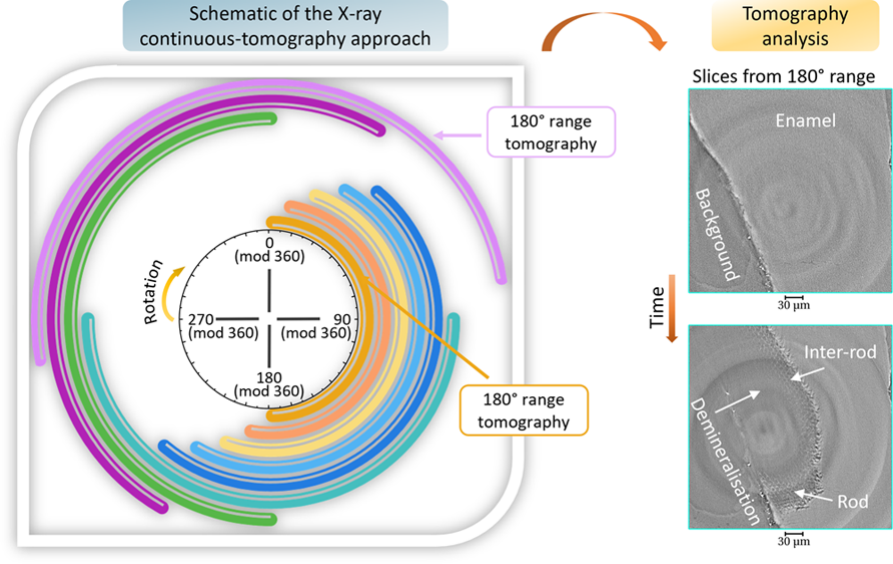

High-resolution spatial and temporal analysis and 3D visualization of time-dependent processes, such as human dental enamel acid demineralization, often present a challenging task. Overcoming this challenge often requires the development of special methods. Dental caries remains one of the most important oral diseases that involves the demineralization of hard dental tissues as a consequence of acid production by oral bacteria. Enamel has a hierarchically organized architecture that extends down to the nanostructural level and requires high resolution to study its evolution in detail. Enamel demineralization is a dynamic process that is best investigated with the help of *in situ* experiments. In previous studies, synchrotron tomography was applied to study the 3D enamel structure at certain time points (time-lapse tomography). Here, another distinct approach to time-evolving tomography studies is presented, whereby the sample image is reconstructed as it undergoes continuous rotation over a virtually unlimited angular range. The resulting (single) data set contains the data for multiple (potentially overlapping) intermediate tomograms that can be extracted and analyzed as desired using time-stepping selection of data subsets from the continuous fly-scan recording. One of the advantages of this approach is that it reduces the amount of time required to collect an equivalent number of single tomograms. Another advantage is that the nominal time step between successive reconstructions can be significantly reduced. We applied this approach to the study of acidic enamel demineralization and observed the progression of demineralization over time steps significantly smaller than the total acquisition time of a single tomogram, with a voxel size smaller than 0.5 μm. It is expected that the approach presented in this paper can be useful for high-resolution studies of other dynamic processes and for assessing small structural modifications in evolving hierarchical materials.

## Introduction

Dental caries is a major worldwide health issue.^1^ Despite the many efforts to prevent and minimize its effects, it remains a challenge to obtain full understanding of the damage occurring in the affected dental hard tissues because of the complex, multi-level spatial architecture of the enamel structure.^2^ There have been very few and limited studies devoted to the visualization of the dynamic process of enamel demineralization with high spatial and temporal resolution. The primary objective in this study was to evaluate the feasibility of synchrotron *in situ* data acquisition using continuous rotation to achieve greater temporal resolution. This methodology is expected to have significance for the study of enamel, including remineralization strategies and biomimetic materials development.^3,4^

Enamel is an acellular tissue that forms the outer layer of tooth and is composed mainly of calcium and phosphorus minerals^5,6^ formed from hydroxyapatite (HAp) crystallites arranged as a hierarchical structure from macroscopic down to the nanoscale.^3,7−10^ Enamel has some remarkable mechanical properties, but it lacks resistance to acids (acid erosionor due to caries).^3,11−13^ When exposed to acid, enamel undergoes dissolution of its structure to form voids that can be visualized at the macro scale down to the microscale (rods and inter-rods) as changes in structure and finally at the nanoscale with the modification of the crystallites and small-angle X-ray scattering (SAXS) signature.^7,10,14−17^ This demineralization process has been shown to be inhomogeneous, with the existence of preferential sites of dissolution,^10,17^ and appears to be a dynamic process with loss, as well as redeposition, of ions.^16,18−20^

Despite numerous studies of the dissolution of enamel characterized using a variety of techniques,^7,14,15,17,21−23^ including detailed study at the nanoscale,^15,23^ there has been limited assessment of the submicron evolution of demineralization and remineralization in 3D. Most of those studies were carried out analyzing samples *ex situ* after natural or artificial demineralization^7,10,21,24−26^ (for a general introduction to the subject, see a recent systematic review on the 3D analysis of human dental caries^14^). With high-resolution tomography, rods and inter-rods structures and 3D demineralization in carious enamel were characterized at submicron resolution (0.325 μm),^27,28^ thereby bringing new insights into the evolution of the enamel structure and localized structural variations, as well as providing inputs for finite element modeling.^24^ However, such an approach lacks information regarding the history of the process, which is essential to understand the pathways of acid dissolution within the enamel structure, to obtain better understanding of the mechanism of dissolution, and potentially to assist with devising new remineralization strategies. In addition, a few studies have looked into the dynamics of demineralization.^27,28^ Time-lapse examination of enamel demineralization has been presented using tomograms^28^ and also carried out in dentine, thereby showing the evolution of the tubular structure during process.^29^ These analyses led to time-lapse visualization of demineralization from independent tomograms, but missed intermediate details.

**Figure 1:**
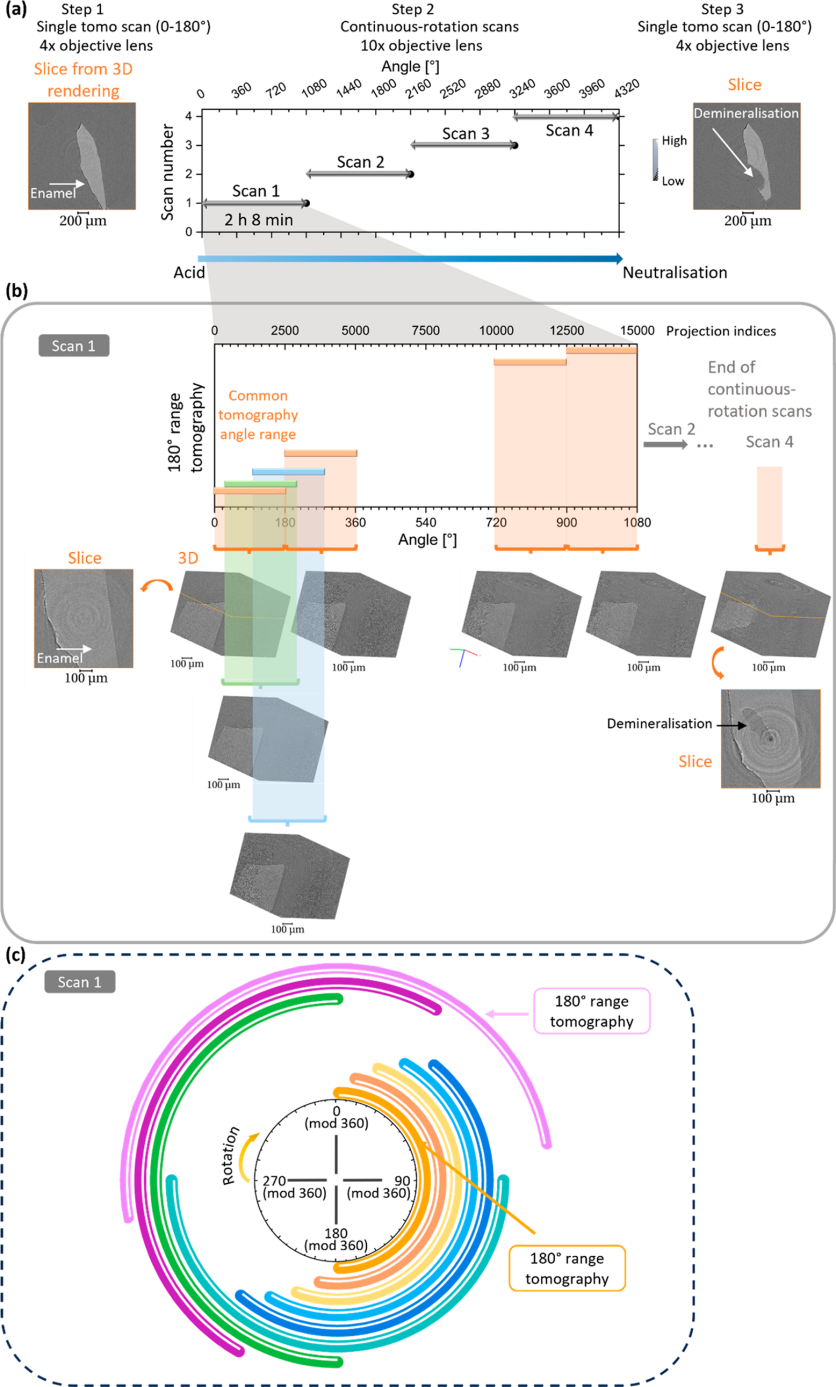
Schematic illustration of the continuous-tomography analysis of the enamel. (a) Overview of the analysis carried out with the iterations corresponding to a new saved scan file as a function of the index of projections (equivalent to the angle of rotation). Image of one horizontal slice of the sample before and after the scan iterations acquired with the 4× objective lens and a voxel size of 0.8125 μm (tomography scan referred to as “tomo”) in contrast with the 10× objective lens used during the iterations under acid immersion before neutralization. Each scan file covered a range from 0 to 1080° and is further described in (b). The 180° scans were extracted to reduce to the size of data sets for analysis. (b) The plot of the angle and projections as a function of tomogram (over the range of 180°) for one scan iteration that contains projections indexed from 0 to 15 000 (equivalent to six consecutive tomograms every 180°) and took around 2 h 8 min. The 3D reconstructed data sets were generated by extracting 2501 consecutive projections, which were then reconstructed to provide 3D details—this is shown with the 3D rendering of some reconstructed data sets with a voxel size of 0.325 μm. Dark- and flat-field images were acquired at the beginning of Scan 1. From the 3D reconstructed volume, slices were selected to illustrate the evolving structural changes seen in enamel from Scan 1 to Scan 4 after immersion in citric acid. (c) Schematic of the analysis of the tomograms from the Scan 1 with the associated rotation angles. This highlighted the overlap of tomography ranges used for the reconstruction of tomogram, which led to an improvement in temporal resolution.

There are research papers describing fast modes of data acquisition that can capture and reveal these intermediate details.^30−32^ To improve the time resolution in the analysis of dynamic events, another approach was reported where continuous rotation scans were used to extract intermediate 3D data sets.^33−36^ This resulted in a high temporal resolution, as demonstrated in a study of foam^34^ where a spatial resolution of 2–3 μm was achieved. For studying smaller structures, such as enamel rods, one requires a fast acquisition time, high resolution, large photon flux, and certain flexibility in the positioning and motion of the sample within its environment. Synchrotron setups can be adapted to accommodate for these challenges.^3,37^ Using the promising new approach and setup using continuous rotation and analysis algorithm applied at different stages of the process, the degradation of enamel was studied in this paper, with the resolution limited by the voxel size of the setup used. Here, a setup that allowed continuous rotation was used with a large field of view at the voxel size of 0.325 μm, as summarized in Figure 1.

## Methods

### Tooth—Optical Image

A sample from a human third molar extracted for noncaries-related therapeutic reasons was used in the study (National Research Ethics Committee; NHS-REC reference 09.H0405.33/Consortium Reference BCHCDent332.1531.TB). The sterilization and preparation of the sample was similar to that described in the previous work.^10,28^ A block of enamel with a thickness of around 400 μm was varnished with commercially available nail varnish, though an exposed window was left, and stored in phosphate-buffered saline. The sample was imaged using optical profilometry [Alicona profilometer (Bruker, U.K.)] prior to demineralization to locate the window. The sample was then glued to a flow cell and immersed in phosphate-buffered saline for storage and transport prior to the tomographic scans.

### Tomography Analysis

The enamel demineralization was monitored using X-ray synchrotron tomography. With uninterrupted processes occurring on the enamel, an improvement in the temporal resolution between tomograms was developed and is summarized in Figure 1. Synchrotron tomography analysis was carried out on Beamline I13-2 at the Diamond Light Source.^38^ The tooth sample was placed in a flow cell mounted on a rotation stage (Figure S1).^28,39^ The experiment was carried out at room temperature using pink beam with a photon energy distribution centered around 25 keV. Two objective lenses were used, 4× and 10×, which provided a voxel size of 0.812 and 0.325 μm and a field of view of 2.1 × 1.8 and 0.83 × 0.7 mm, respectively (total duration of a single tomography scan was approximately 24 min with an exposure time of 0.5 s). The rotation stage enabled an unlimited, continuous rotation of the sample. Single (over the range of 180°) and continuous-rotation tomographic scans were performed. During a single tomography scan, the rotation stage was moved from 0° to 180°. In contrast, the rotation stage moved from 0° to an integer multiple of 360° when a continuous-rotation scan was run. To improve temporal resolution of the data acquisition, the sample was scanned continuously while rotating. This generated a large data set of 15 001 images, which were acquired in 2 h 8 min. To speed up the process of analyzing this large data set, a number of smaller data sets were extracted from it. This was equivalent to six tomograms of 2501 projections, which were acquired each over the 180° angular range. The full analysis was carried out with scripts written in-house at I13, which allowed a set of 2501 projections to be extracted and reconstructed. Before launching a continuous-rotation scan, 20 dark- and 20 flat-field images were acquired for background correction. For the continuous scan, the acquisition was carried out using an objective lens of 10×, an angular step of 0.072°, and an exposure time of 0.5 s. Separate single tomography scans were performed using the objective lens of 4× (before and after the enamel treatment), the angular step of 0.072°, and the exposure time of 0.5 s to record 40 dark- and 40 flat-field images, while for the 10× objective lens before the treatment the angular step was 0.1° and the exposure time was 0.5 s.

The enamel sample was immersed in artificial saliva (0.7 mmol L^–1^ CaCl_2_, 0.2 mmol L^–1^ MgCl_2_, 4.0 mmol L^–1^ KH_2_PO_4_, 30.0 mmol L^–1^ KCl, 20.0 mmol L^–1^ HEPES pH 7),^40^ then immersed in citric acid pH 2.2 (scanning was done for 8 h 30 min of immersion), and finally in artificial saliva. While this demineralized enamel, it was not a physiological representation of the oral environment but provided preliminary details and methods. For 180° rotation scans, extraction of intermediate data sets and reconstruction were done using Savu software and I13-2 Python scripts.^10,28,41−43^Figure 1 shows the details of one continuous-rotation acquisition and subsequent volume reconstruction of the tomography data, which highlights the results achieved by the extraction of intermediate tomograms (Figure 1b). The projection images, 3D renderings of synchrotron data, were analyzed using DAWN;^44^ ImageJ-Fiji;^45,46^ Avizo packages, as described in our previous papers;^10,14^ and CorelDRAW software.

### SEM imaging

After synchrotron tomography analysis, the sample was placed on SEM holder and imaged using secondary and backscattered electron images (SEi and BSi) with a SEM Tescan Lyra 3 (Tescan, Czech Republic) and an accelerating voltage of 5 keV to visualize the exposed window after demineralization.

## Results and Discussion

### Optical Microscopy

**Figure 2:**
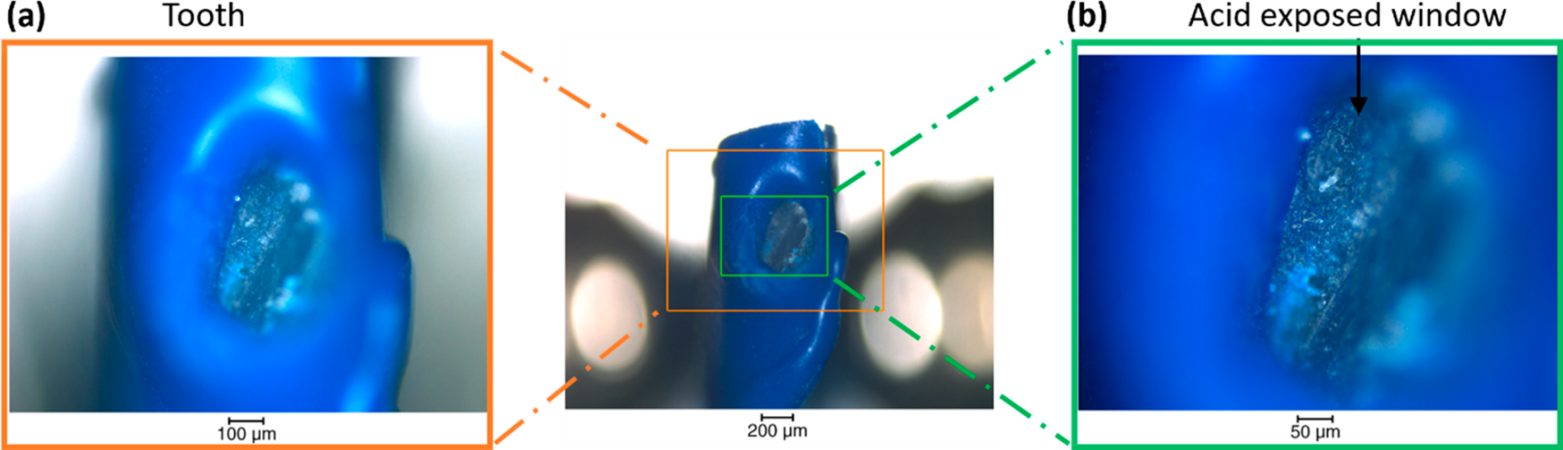
Optical images of the sample before synchrotron analysis. Images of the tooth sample covered with blue varnish with magnified region in (b) showing the window left for the demineralization of the enamel.

Figure 2 shows the sample before the synchrotron experiment and provides the dimensions and localization information on the exposed window of the sample. In Figure 2b, an unexposed region is observed, which is used for the acid exposure.

### Synchrotron Tomography

**Figure 3:**
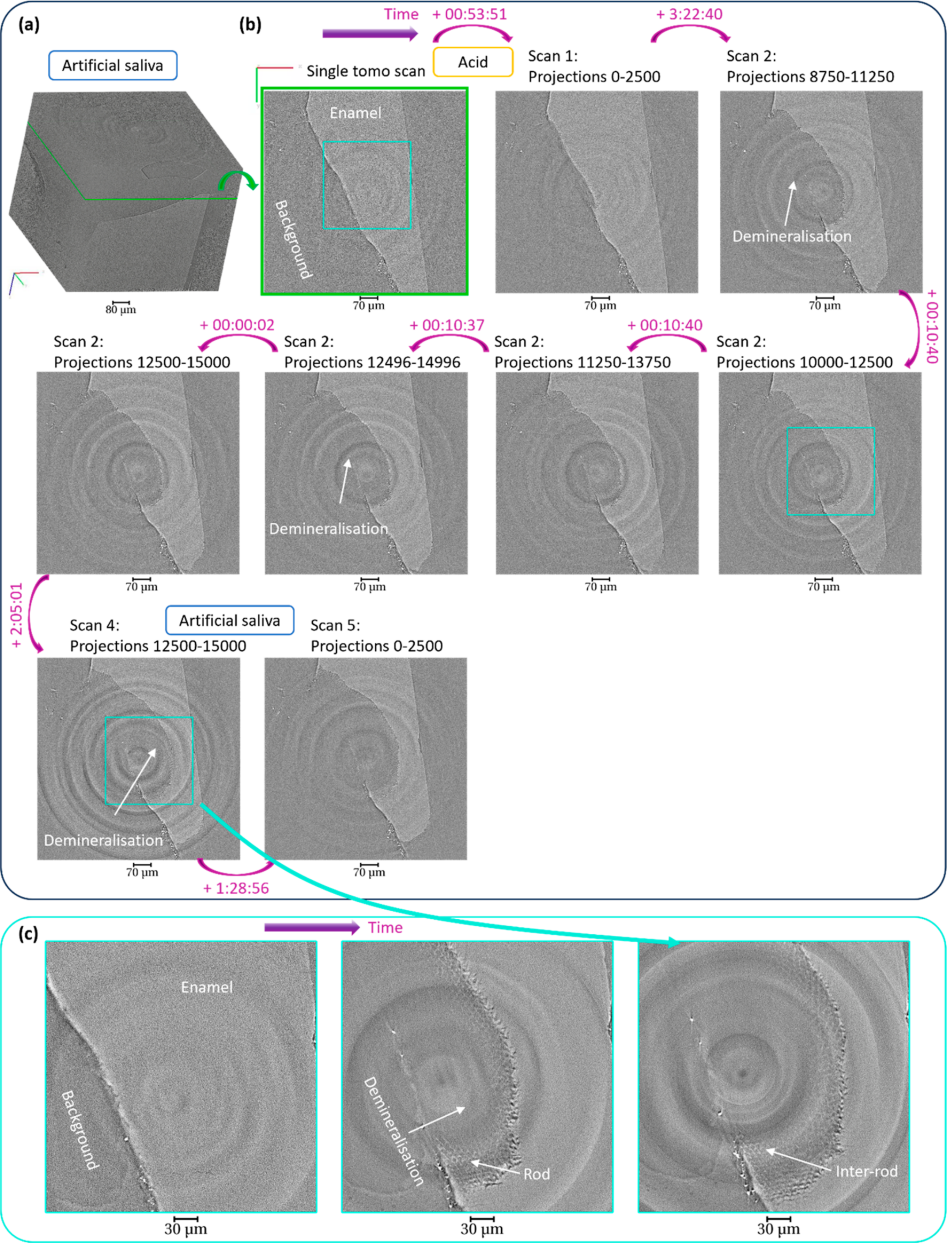
Progress of demineralization of enamel over a time period, captured using synchrotron X-ray tomography. (a) 3D rendering of the enamel before demineralization and highlighting of the position of a slice. (b) Visualization of the progression of the demineralization with the slice described in (a) and sequence of slices (generated from 3D reconstruction) at different events during demineralization of enamel with details of the projections used to reconstruct the data set (see Figure 1 for the details of the file and projections) and the time. The time difference (in the format hh:mm:ss) corresponds to the duration between the starting time of the acquisition of the first projection of two tomograms with the description of the slices. (c) 2D images of regions of interest (1080 × 1080 × 2110 pixels) highlighted with blue boxes in the slices in (b) with the details of the rods and inter-rod substance. The data sets were reconstructed with a voxel size of 0.325 μm.

**Figure 4:**
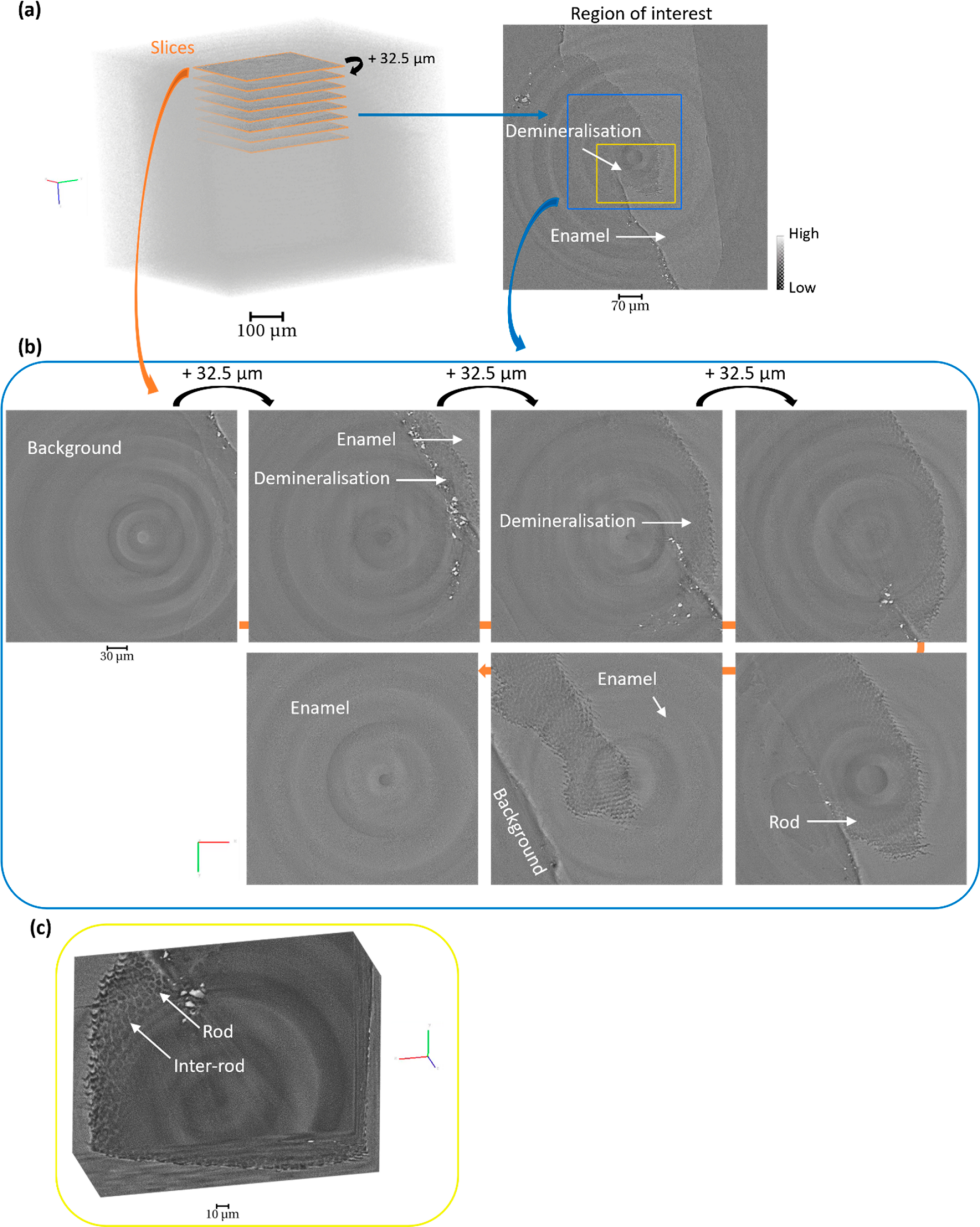
Evolution of the structure of the enamel through the cavity in a tomogram. (a) 3D rendering of the enamel with the highlight of slices through the thickness of the sample. Two regions of interest are illustrated on a slice located in the demineralized region and described in (b,c). (b) Sequence of slices with separation distances of 32.5 μm along the depth of the tomogram from a region of interest (1080 × 1080 × 2110 pixels) showing the demineralized region within the sample, blue region. (c) Magnified region described in (a,b) with the 3D rendering of a region of interest (in yellow) showing the enamel, rods, and the demineralized region (745 × 552 × 300 pixels). The tomography data sets were reconstructed with a voxel size of 0.325 μm.

The tooth sample was immersed in liquid solutions and scanned by using synchrotron X-ray tomography. Continuous rotation of the sample was performed, and the liquid solutions were changed after the scan iteration to neutralize the acid. The reconstruction of the tomography data was carried out by using different sets of projections acquired during the experiment. Figure 3 summarizes the results obtained at different time points and under different solutions. From the analysis of the data and visualization of the slices with detailed enamel structure, this demonstrates the possibility to reconstruct and visualize enamel structure for different time points using intermediate sets of tomograms. This is illustrated using the horizontal slices generated from the set of tomograms with time (Figure 3b). The analysis provides an approach to select a tomogram (over the range of 180°) at various time points of the experiment. The progression of the demineralization was identified from the variation of gray value (pixel intensities) and the loss of material on the sample after acid immersion in comparison with the initial data set. The region of the demineralization is highlighted in three data sets with clear observation of the modification of the enamel with time and the details of the rod enamel structure (Figure 3c). The possibility to have 3D data sets is also illustrated in Figure 4 with the view of the demineralization in enamel within the depth of the sample at a time point. The anisotropy of the demineralization was observed with detailed information on the structure of the enamel (Figure 4 and Figure S2) in agreement with previous studies.^10,28,47^

### SEM Imaging

**Figure 5:**
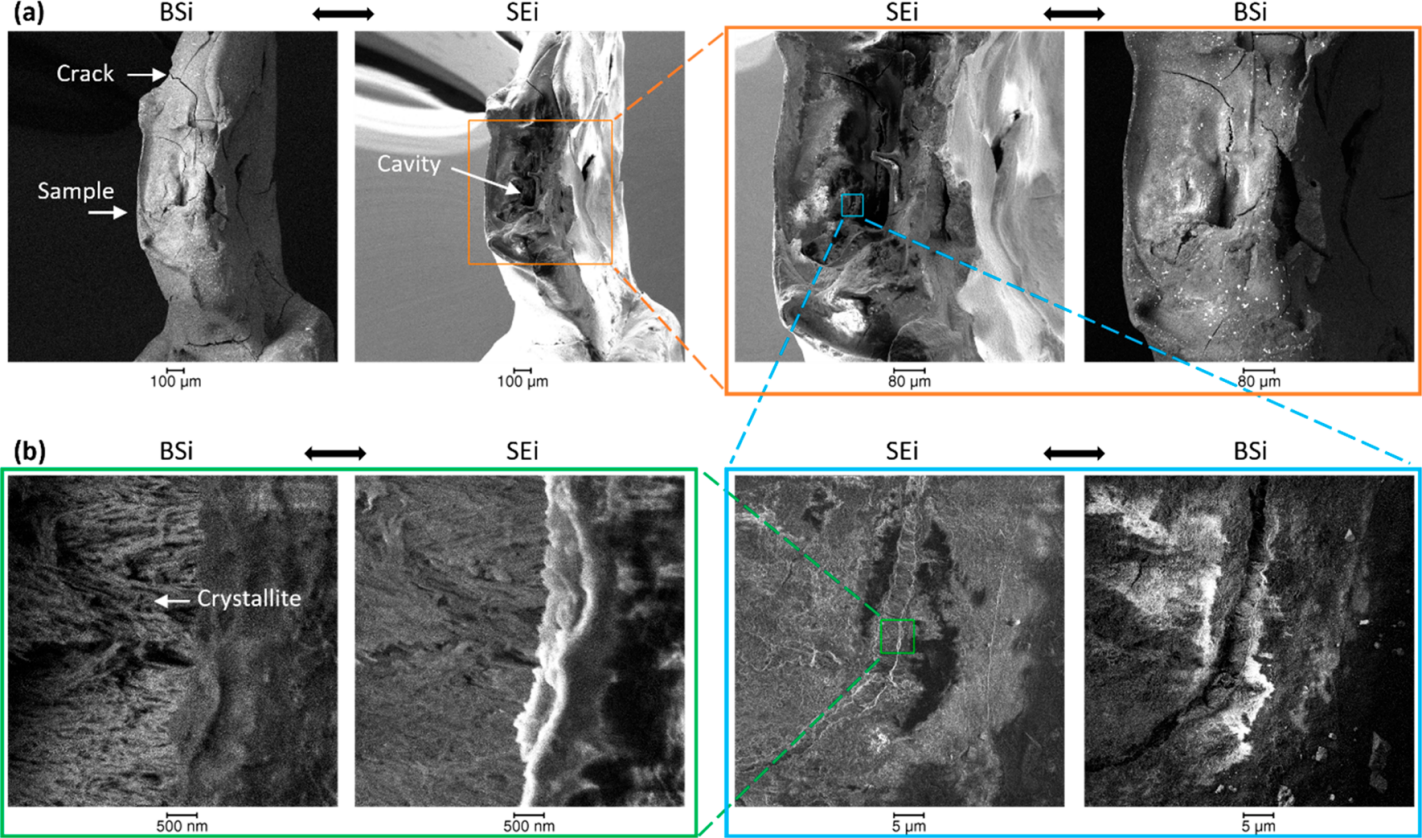
SEM images of the sample after X-ray tomography. (a) SEi and BSi analysis at various magnifications of the enamel showing the demineralized region and overall damages on the sample and (b) high-magnification image.

Figure 5 shows the SEM images acquired on the sample, thereby providing the topology of the structure. Significant damages were seen and suggested to arise from the demineralization of the sample. By zooming into the cavity, the crystallites could be visualized (Figure 5b), which was a consequence of the demineralization, as noted before.^7^ The sample cracks were likely to arise from drying in vacuum during the analysis, as these were not seen optically before the experiment (Figure 2).

## Conclusion

The importance of developing methods for identifying and visualizing dynamic changes in dental enamel during demineralization was reported. In situ X-ray synchrotron tomography was performed while enamel was exposed to a citric acid solution of pH 2.2. The continuous rotation of the sample and processing of the data sets allowed for a sequence of fast 3D scans to be carried out that demonstrated structural modifications of enamel after exposure to acid. Alteration of the enamel structure was detected at different time points and could be localized with a voxel size down to 0.325 μm and a time frame of 0.5 s possible with intermediate tomograms. This approach can be used to locally track variations of enamel structure with high resolution so as to provide an advanced perspective on the analysis and can also be extended to study the action of bacteria products on enamel at the resolution of the beamline used. It also opens a large variety of new applications with biological significance to study biomimetic materials, as well as other fields of research, where it is important to understand how and where materials modifications take place.

## Data Availability

The data collected and interpreted in this study are maintained by the authors and can be made available upon request.

## Supporting Information Available

Two figures of experimental results (PDF)
