# Supplementary material for: Advanced Time-Stepping Interpretation of Fly-Scan Continuous Rotation Synchrotron Tomography of Dental Enamel Demineralization
Source: Chem Biomed Imaging. 2024 Feb 8;2(3):213–21. doi: 10.1021/cbmi.3c00121 (PMC10966730; doi:10.1021/cbmi.3c00121)
Supplement: Supplementary file 1 — im3c00121_si_001.pdf [file im3c00121_si_001.pdf]

Supporting Information

Advanced time-stepping interpretation of fly-scan continuous rotation synchrotron tomography of dental enamel demineralisation

Authors

Cyril Besnard*[a], Ali Marie[a], Sisini Sasidharan[a,1], Shashidhara Marathe[b], Kaz Wanelik[b], Robert A. Harper[c], Christoph Rau[b], Richard M. Shelton[c], Gabriel Landini[c], Alexander M. Korsunsky[d*]

[a] Department of Engineering Science, University of Oxford, Parks Road, Oxford, Oxfordshire, OX1 3PJ, U.K.

[b] Diamond Light Source Ltd., Didcot, Oxfordshire, OX11 0DE, U.K.

[c] School of Dentistry, University of Birmingham, 5 Mill Pool Way, Edgbaston, Birmingham, West Midlands, B5 7EG, U.K.

[d] Trinity College, University of Oxford, Broad St, Oxford, Oxfordshire, OX1 3BH, U.K.

Email addresses:

cyril.besnard@eng.ox.ac.uk, ali.marie@eng.ox.ac.uk, sisini.sasidharan@eng.ox.ac.uk, shashidhara.marathe@diamond.ac.uk, kaz.wanelik@diamond.ac.uk, R.A.Harper@bham.ac.uk; christoph.rau@diamond.ac.uk, R.M.Shelton@bham.ac.uk, G.Landini@bham.ac.uk, alexander.korsunsky@eng.ox.ac.uk

* ***Corresponding authors***: Cyril Besnard, cyril.besnard@eng.ox.ac.uk

Alexander M. Korsunsky, alexander.korsunsky@eng.ox.ac.uk

Present address:

[1] Department of Materials, Imperial College London, SW7 London, U.K.

Supporting Information (SI)

Table of Contents

Figures

Figure S1. p. 3

Figure S2. p. 4

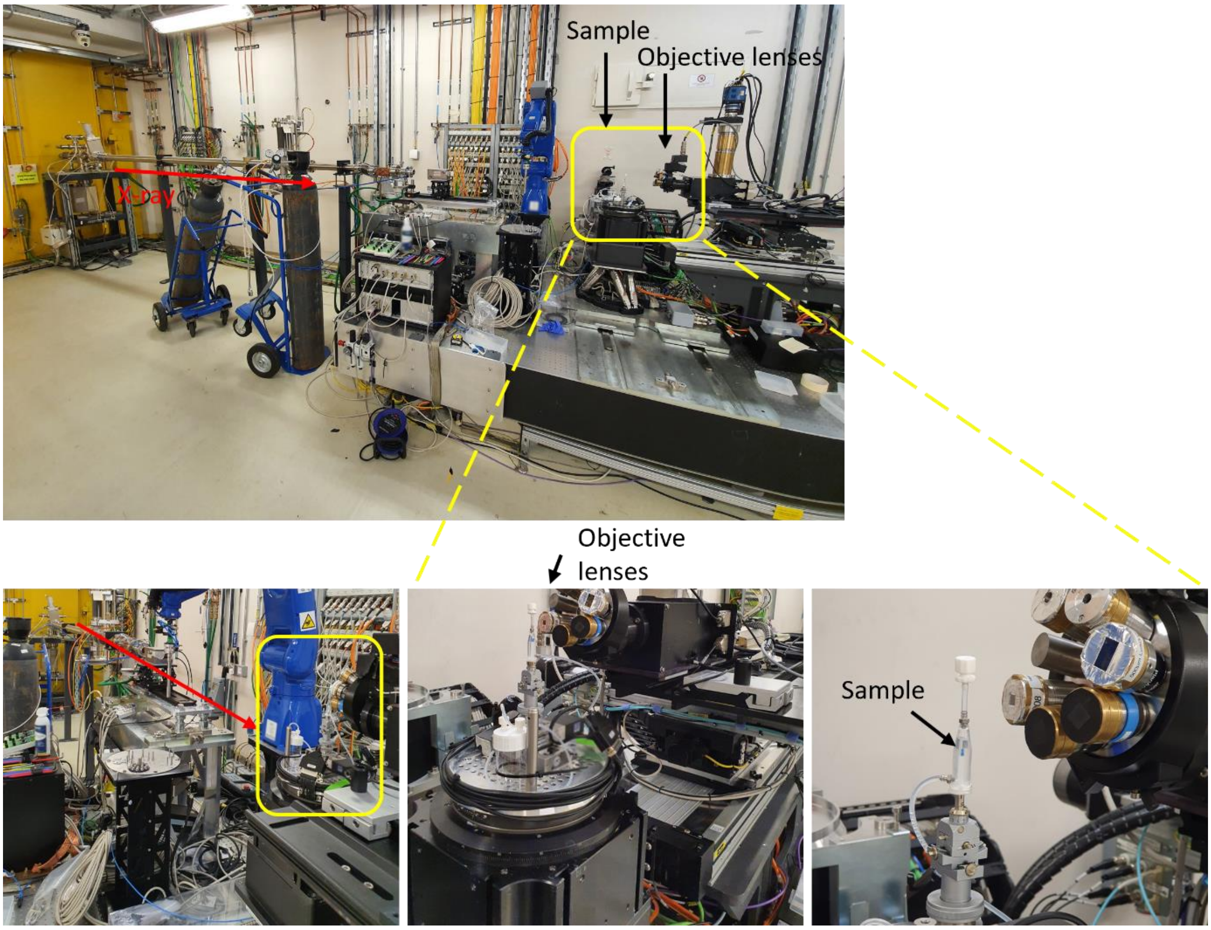

Figure S1. Setup of the experiment on the beamline I13-2 (Diamond Light Source Ltd., Didcot). Photographs of the sample positioned on the tomography stage, of the objective lenses.

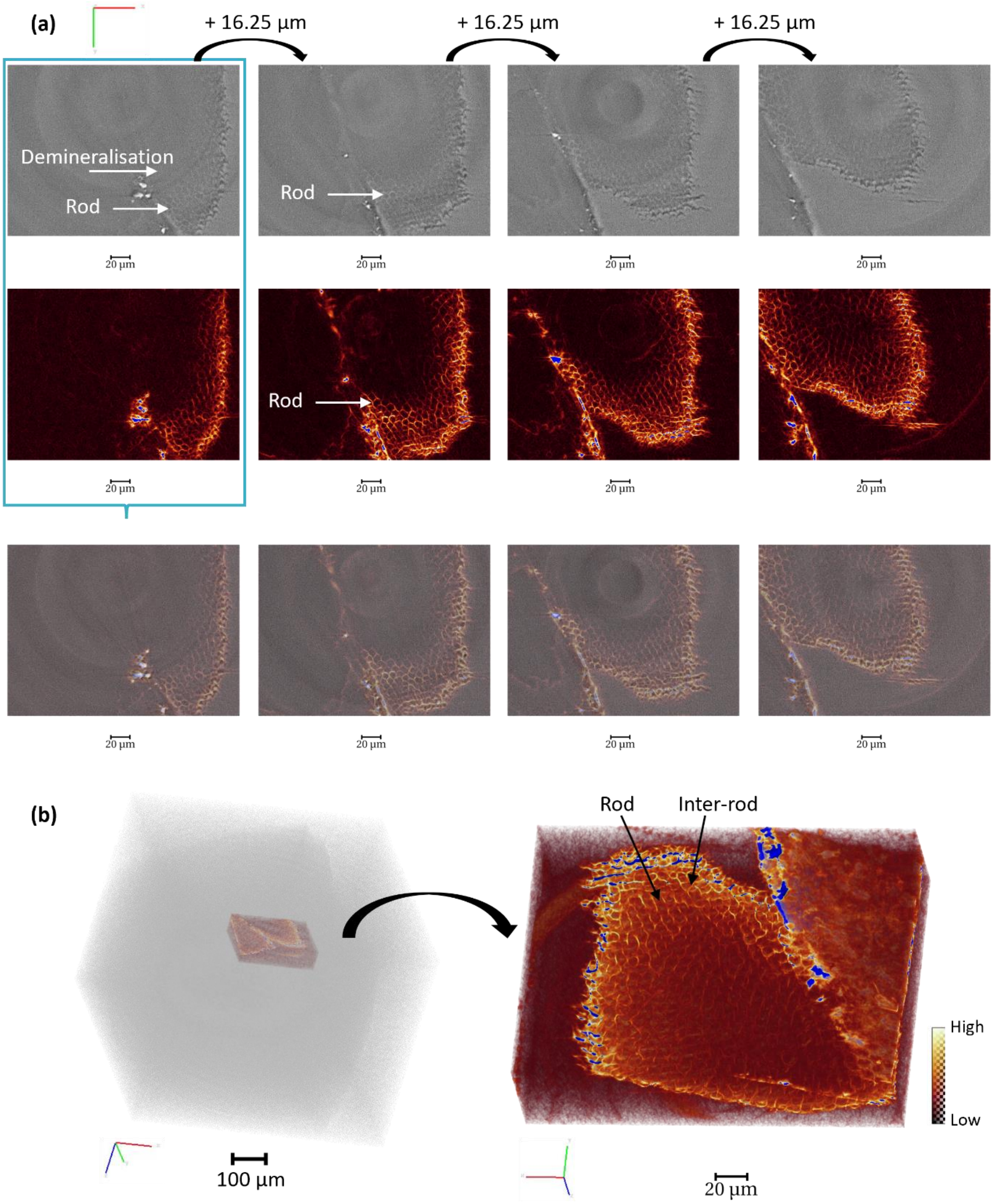

Figure S2. Image analysis of the tomography dataset. (a) Slices from the non-filtered dataset at different depths in the region of interest extracted from one time point (745 × 552 × 300 pixels), described in Figure 4. Slices after filtering using membrane enhancement filter following analysis in the previous works[1,2]. Superposition of the two datasets. (b) 3D rendering of the large dataset with the view of the 3D rendering of the filtered dataset, a voxel size of 0.325 µm. The structures of the rods and inter-rod substances were highlighted on the Figure.

References

1 Besnard, C., Marie, A., Sasidharan, S., Harper, R. A., Marathe, S., Moffat, J., Shelton, R. M., Landini, G. & Korsunsky, A. M. Time-lapse in situ 3D imaging analysis of human enamel demineralisation using X-ray synchrotron tomography. *Dentistry Journal* **11**, 130, doi:https://doi.org/10.3390/dj11050130 (2023).

2 Besnard, C., Marie, A., Buček, P., Sasidharan, S., Harper, R. A., Marathe, S., Wanelik, K., Landini, G., Shelton, R. M. & Korsunsky, A. M. Hierarchical 2D to 3D micro/nano-histology of human dental caries lesions using light, X-ray and electron microscopy. *Materials & Design* **220**, 110829, doi:https://doi.org/10.1016/j.matdes.2022.110829 (2022).
